# Improving the Thermal Stability of Hydrophobic Associative Polymer Aqueous Solution Using a “Triple-Protection” Strategy

**DOI:** 10.3390/polym11060949

**Published:** 2019-06-01

**Authors:** Bo Yang, Jincheng Mao, Jinzhou Zhao, Yang Shao, Yang Zhang, Zhaoyang Zhang, Qingye Lu

**Affiliations:** 1 State Key Laboratory of Oil and Gas Reservoir Geology and Exploitation, Southwest Petroleum University, Chengdu 610500, China; bo.yang1@ucalgary.ca (B.Y.); 201722000463@stu.swpu.edu.cn (Y.S.); 201521000202@stu.swpu.edu.cn (Y.Z.); 201611000102@stu.swpu.edu.cn (Z.Z.); 2 Department of Chemical and Petroleum Engineering, University of Calgary, Calgary, AB T2N 1N4, Canada

**Keywords:** high temperature resistance solution, hydrophobically-modified polyacrylamide, hydrophobic associative water-soluble polymers, high temperature responsive crosslinking agent, fracturing fluid

## Abstract

Because of their high viscoelasticity, Hydrophobic Associative Water-Soluble Polymers (HAWSPs) have been widely used in many industrial fields, especially in oilfield flooding and fracturing. However, one major problem which limits the wide applications of HAWSPs is their weak resistance to high temperatures. Once the temperature increases over 100 °C, the viscosity of the fracturing fluid decreases rapidly, because high temperatures reduce fluid viscosity by oxidizing the polyacrylamide chains and weakening the association of hydrophobic groups. To improve the high temperature resistance of one HAWSP, a triple-protection strategy was developed. First, rigid *N*-vinyl-2-pyrrolidone moiety was introduced into the polymer chains. Second, an environmentally-friendly deoxidizer, carbohydrazide, was selected to prevent polymer oxidization by scavenging dissolved oxygen. Results showed that both the rigid groups and the deoxidizer improved the temperature resistance of the polymer and helped it maintain high viscosity under high temperature and shear rate. Using these two protection strategies, the resistant temperature of the polymer could reach 160 °C. However, the polymer network still got severely damaged at further elevated temperatures. Therefore, as the third protection strategy, the pre-added high temperature responsive crosslinking agent was applied to form new networks at elevated temperatures. The results have shown that the optimized polymer solution as a kind of fracturing fluid showed good temperature resistance up to 200 °C.

## 1. Introduction

Hydrophobic Associative Water-Soluble Polymers (HAWSPs) or Hydrophobically-Modified Polyacrylamide (HMPAM) derivatives are polymers that contain a small number of hydrophobic groups grafted onto the hydrophilic polymer backbone. The aggregation of hydrophobic groups in aqueous solution forms a network structure, resulting in a sharp increase in viscosity. Due to their good thickening ability, HAWSPs have been widely used in many aspects of oilfield operations [1]. especially in polymer flooding and hydraulic fracturing [2,3,4,5,6,7]. For example, HAWSPs are employed as one type of popular thickeners which can effectively carry sand without adding any crosslinking agents for fracturing application [4,5,6]. In addition, the polymers themselves are completely soluble; hence, after gel breaking, formation damage caused by gel residue is very low (permeability reduced by less than 10%) [6].

However, at high temperature, the viscosity retention capability of these polymers is very poor, which greatly limits their application for high temperature environments. Temperature and oxygen are two major factors leading to such decrease of viscosity [8,9]. On one hand, high temperature leads to chain breakage, which is the main reason for reducing the viscosity of the solution in the absence of cations, and on the other hand, accelerates the hydrolysis of amide groups, although this might not necessarily reduce the viscosity. Actually, the viscosity of polyacrylamide solution increases with hydrolysis when the degree of hydrolysis is less than 40–50% [10], then viscosity begins to decrease. When the hydrolysis degree reaches 70%, the viscosity is about the same as that of 20% hydrolysis degree [11].

Oxygen leads to oxidative degradation by breaking polymer chain backbone. It has been reported that a small amount of dissolved oxygen can cause a significant decrease in the viscosity of the polyacrylamide solution, and with increasing temperature, the degradation can be radically accelerated [12]. Seright et al. (2010) showed that, in the absence of dissolved oxygen and divalent cations, the polymer backbone can remain stable to maintain at least half of its original viscosity for more than 8 years at 100 °C and for approximately 2 years at 120 °C [13].

Deoxidizers have been intensively studied in various industries, for example, deoxygenation and corrosion prevention of boilers. Commonly used deoxidizers can be classified into inorganic and organic types. Inorganic deoxidizers include sodium sulfite, sodium thiosulfate, hydrazine, etc. Organic deoxidizers include carbohydrazide, acetaldehyde oxime, acetone oxime, D-isoascorbic acid, sodium D-isoascorbate, and so on [14]. In order to overcome the environmental disadvantages of surfur-containing oxygen scavengers which can produce toxic gases at high temperature and hydrazine scavengers itself is strongly toxic, carbohydrazide, acetone oxime, sodium D-isoascorbate, and other environmentally-friendly agents have been widely used. Use of deoxidizers to improve the high temperature resistance of fracturing fluids has long been studied. For example, Pakulski and Gupta (1994) improved the temperature resistance of guar fracturing fluids to 150 °C by adding methyl ethyl oxime [15]. Gupta and Carman (2010) further showed that chalcogen heterocyclic compounds containing oxygen or sulfur are helpful for extending the high temperature effectiveness of aqueous gels [16]. It was found that these compounds can prevent the thermal degradation of gels at temperatures as high as 205 °C. The sterically unhindered oxygen atom on the heterocyclic compound carries two unshared pairs of electrons, which can be donated and reduce oxygen free radicals to maintain the gel stability. But some reducing agents can lead to faster degradation of the polymer chains [12], so it is necessary to investigate whether a deoxidizer is compatible with the acrylamide-based polymers. 

The other reason for viscosity loss of HAWSPs is that hydrophobic interaction decreases with increasing temperature which results in a weakening or even a suppression of the association at high temperature. Changing the length of hydrophobic monomer chains, hydrophobic monomer content and its distribution in the polymer chain could increase the viscoelasticity of the solution at low and moderate temperatures [17,18,19,20,21]; however, it is difficult to improve the performance of these polymers at high temperature. Thus, some thermostable monomers, such as 2-acrylamido-2-methyl propane sulfonic acid (AMPS) or *N*-vinyl-2-pyrrolidone (NVP), were introduced into the linear acrylamide-based polymer to improve the temperature resistant performance. Furthermore, metal crosslinking agents, such as zirconium, were used to increase the viscoelasticity of the solution. The modified polymer was reported to be used in fracturing fluids for formations at over 200 °C [22,23,24,25,26]. However, this crosslinking technology needs to be carried out under specific pH conditions, for example, pH usually is 3–5 when adopting zirconium-based crosslinkers, which increases the operation difficulty and equipment requirements. 

Polyethyleneimine (PEI) can be used as a crosslinking agent which can react with amide groups to form covalent bonds at high temperature to form network structures. It has been widely used in chemical flooding and water shutoff operations in oilfields [27,28,29,30,31]. In our previous studies, the gel system of PEI cross-linked partially hydrolyzed polyacrylamide was successfully applied to water shutoff treatments [32,33]. The crosslinking reaction rate between PEI and amide groups is mainly dependent on temperature, which could take several days in low or moderate temperature reservoirs [27,28,29,30,31,32], this timeframe cannot meet the requirements of fracturing operations in terms of rapid crosslinking. PEI has been employed to crosslink acrylamide-based polymer fracturing fluids in high temperature situations as high temperature can drastically accelerate the reaction [34,35]. For example, at 160 °C, the crosslinking time can be shortened to about 20 min; however, this time frame still makes it difficult for low viscoelastic fluids to meet industrial requirements. In order to keep the viscosity of the fluids consistently higher than the industrial standard, zirconium can be added for co-crosslinking [36].

**Figure 1:**
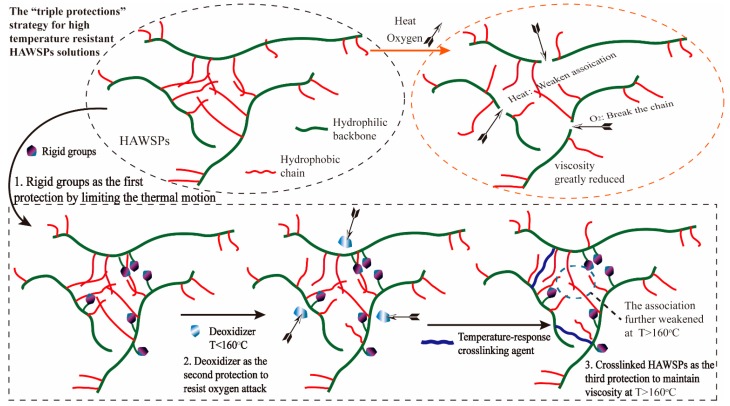
Schematic of the “triple-protection” strategy to enhance the high temperature resistant performance of hydrophobic associative water-soluble polymers (HAWSPs).

**Figure 2:**
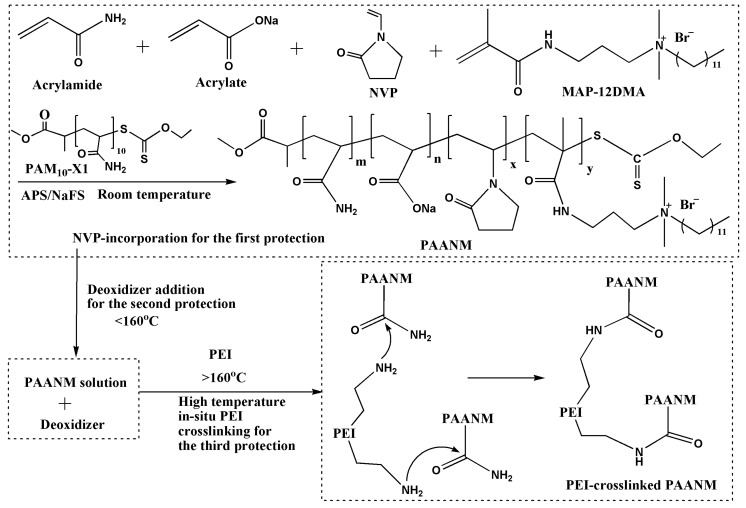
Schematic of the synthesis of *N*-vinyl-2-pyrrolidone (NVP)-incorporated HAWSPs (named as PAANM) by redox-initiated aqueous polymerization where the rigid moiety NVP works as the first protection to limit thermal motion in HAWSPs, followed by the addition of environmentally-friendly deoxidizer into the polymer solution as the second protection, and polyethyleneimine (PEI) as a crosslinking agent to react with amide groups to form covalent bonds at high temperature in HAWSPs as the third protection.

Our objective was to develop a triple-protection strategy to tackle the challenge of improving high temperature resistance of a HAWSP, which is schematically demonstrated in Figure 1 and Figure 2. First, NVP was added during the polymerization as a rigid group to increase the rigidity of the polymer chains, which can limit their thermal movement at elevated temperatures in order to achieve higher viscosity retention. Secondly, an environmentally-friendly deoxidizer was added into the solution to prevent the polymer from being oxidized and degraded. And thirdly, PEI was used to crosslink with the amide groups in the HAWSP to increase the fluids viscosity especially at elevated temperature when the crosslinking can be achieved within minutes. HAWSPs can retain strong viscoelasticity only at low and medium temperature, so, most of the HAWSPs based fracturing fluids can only resist temperatures below 140 °C [37]. With increasing temperature, the hydrophobic association will be weakened to cause viscoelasticity loss, however, the crosslinking of HAWSP with PEI will be significantly expedited to form new network structures to increase the fluids viscosity. Because the new networks are covalently linked, they are expected to have strong resistance to high temperature, avoiding the need of using metal ions for co-crosslinking.

## 2. Materials and Experimental Methods 

### 2.1. Materials

Acrylamide (AM), acrylic acid (AA), ammonium persulfate (APS) and 2,2’-azobis(2-methylpropionamide) dihydrochloride (V-50), sodium bromate (NaBrO_3_) were all purchased from Chengdu Kelong chemical reagents corporation (Chengdu, China). Sodium formaldehyde sulfoxylate dihydrate (NaFS, Kefeng, Shanghai, China), potassium ethyl xanthogenate (PEX, Kefeng, Shanghai, China), methyl-2-bromopropionate (MBP, JK Chemicals, Beijing, China), polyethyleneimine (PEI, Best-reagent, Chengdu, China), and *N*-vinyl-2-pyrrolidone (NVP, Best-reagent, Chengdu, China) were used. *N*-(3-methacrylamidopropyl)-*N*,*N*-dimethyldodecan-1-aminium (MAP-12DMA) was prepared as the hydrophobic functional monomer following the previously reported procedure [37]. Chain transfer agent PAM10-X1 was prepared following the literature method [30]. All chemicals and reagents were of analytical grade and used without purification.

### 2.2. Synthesis of the “Triple-Protection” Enhanced Hydrophobic Associative Water-Soluble Polymers (HAWSPs)

The schematic for the preparation of high temperature resistant HAWSPs is shown in Figure 2. The synthesis of HAWSPs with certain temperature tolerance is the first step to achieve high temperature resistance of polymer solution. The length of hydrophobic block, molecular weight and its distribution play an important role in the temperature resistance of polymers [5,38,39]. In our previous studies [37], the chain transfer reagent PAM_10_-X1 with high chain transfer constant was added to achieve the “living” polymerization and can effectively control molecular weight and its distribution [40]. The hydrophobic block length was easily controlled by changing the dosage of the hydrophobic monomer (MAP-12DMA) in the absence of additional surfactant, because it can dissolve in water and aggregate into micelles automatically. Furthermore, the addition of NVP was expected to improve the rigidity of the polymer. 

The typical synthesis steps are as follows: AA (2.72 g, 37.8 mmol) and NaOH aqueous solution (7.56 mL, 2.5 mol/L) were added into a beaker, with pH around 6–7. Then, AM (9.36 g, 131.7 mmol), NVP (2.1 g, 18.9 mmol), quaternary ammonium cationic monomer MAP-12DMA (0.23 g, 0.6 mmol), PAM_10_-X1 aqueous solution (0.5 mL, 0.2 wt%) and 28.0 mL distilled water were mixed together in a breaker and ultrasonically dispersed for 10 min. 

Then the mixture was gently degassed with argon for 30 min before successively injecting 1.0 mL of 2.0 wt% aqueous solutions of NaFS and APS. The reaction took place at room temperature. After 4 h, colorless to light milky gel was obtained. The gel was cut and washed three times with ethanol (100 mL each time), then it was dried in a vacuum oven and crushed into fine particles.

All polymer solutions were prepared by completely dissolving polymer powders into deionized water under sealed magnetic stirring in a beaker prefilled with nitrogen. Unless otherwise specified, the polymer concentration used was 0.6 wt%. In the screening of deoxidizers, different deoxidizers were pre-placed in a beaker containing dissolved polymer powder. Finally, the “triple-protection” enhanced HAWSPs solution was prepared by simultaneously dissolving the PEI polymer and the deoxidizer in water.

### 2.3. Materials Characterization and Evaluation 

The PAANM polymers were characterized by ^1^H NMR, environmental scanning electron microscope (ESEM), thermogravimetric analysis (TGA), and rheological measurements. The ^1^H NMR spectra were recorded on a Bruker 400 MHz nuclear magnetic resonance (NMR) spectrometer. A PAANM solution with a concentration of 1 mg/mL was prepared in D_2_O and its ^1^H NMR spectrum was recorded at 25 °C. To characterize the microstructure of the polymer fluid, a PAANM solution was cryogenically frozen using liquid nitrogen and examined using an ESEM (Quanta 450, FEI, Hillsboro, OR, USA) operating at an accelerating voltage of 20 kV. The TGA was conducted using the DSC823 Thermal Analyzer (Mettler Toledo, Switzerland). 99.999% N_2_ was purged at 50 mL/min as the protective gas and the polymer was heated from 40 °C to 600 °C at a heating rate of 10 °C/min. The rheological properties were determined using the HAAKE Rheostress-6000 Rheometer (Haake, Karlsruhe, Germany) in a temperature range of 30–200 °C using prepared polymer solutions. Each test was run for 1 to 2 h at the desired temperature under a constant shear rate of 170 s^−1^ in a sealed concentric cylinder. The shear rate is chosen because the standard of petroleum industry in China is to measure the viscosity at 170 s^−1^.The viscoelasticity tests included stress sweep and frequency sweep on a cone plate. The range of shear stress was 10^−3^–10^2^ Pa and the range of frequency was 0.1–10 Hz.

### 2.4. Tests of Materials as Hydraulic Fracturing Fluid

In addition to sand carrying capacity and the extent of causing reservoir damage, the rheological properties at high temperatures are the primary index to evaluate whether a polymer can be used as a fracturing fluid.

The settling velocity of the ceramsite (0.42–0.84 mm in diameter) in the fracturing fluids was used to determine the sand carrying capacity. Briefly, the ceramsite and the fracturing fluids were mixed with a sand ratio of 10–30 *v*/*v*% and placed in a 50 mL graduated cylinder at room temperature. The distance and time all ceramisite took to sink to the bottom were observed and recorded, and the settling velocity was calculated.

Gel breaking test was used to assess whether polymer can be degraded effectively after adding a gel breaker to indirectly evaluate its tendency and extent to cause formation damage. Gel breaker (NaBrO_3_) was added in fracturing fluid and stirred uniformly before high temperature test. The shear rate was 170 s^−1^, and the temperature increased from 30 to 200 °C in 30 min and lasted for two hours.

## 3. Results and Discussion

### 3.1. Characterization of PAANM Polymers 

**Figure 3:**
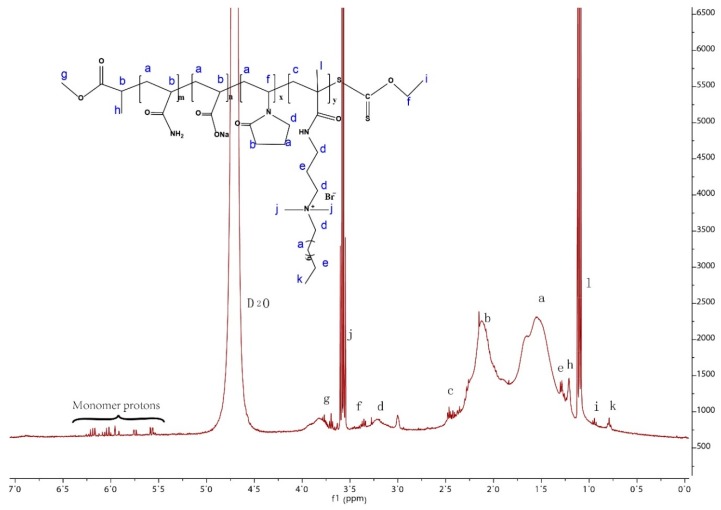
^1^H NMR spectrum of polymer PAANM (10% NVP).

The ^1^HNMR spectrum of the polymer PAANM (10% NVP) is shown in Figure 3. The spectrum displayed the expected strong resonances of the methylene protons (a; *δ* = 1.55 ppm; and b; δ = 2.12 ppm) on the polymeric main chains. The characteristic peaks of the hydrophobic chain are also obvious: methylene protons (d; *δ* = 3.21 ppm), (e; *δ* = 1.29 ppm), methyl protons (j; *δ* = 3.56 ppm), (k; *δ* = 0.79 ppm). Other protons are as follows: (c; *δ* = 2.44 ppm), (f; *δ*= 3.36 ppm), (h; *δ* = 1.21 ppm), (i; *δ* = 0.94 ppm), and (l; *δ* = 1.09 ppm). All these peaks suggest that the designed polymer was successfully obtained. There are a few very small peaks between 5.5 and 6.5 ppm, which may be due to the monomer residues.

**Figure 4:**
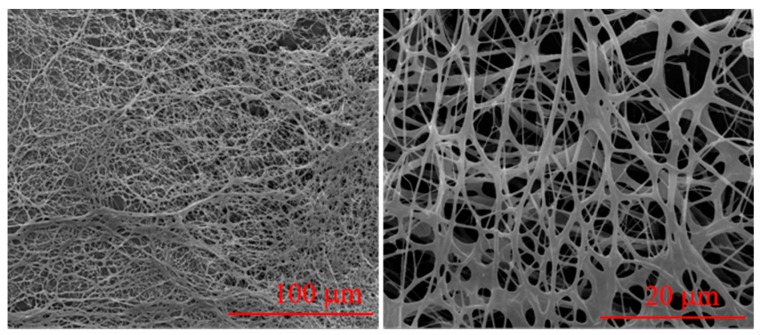
Environmental scanning electron microscope (ESEM) image of the 0.1 wt % PAANM solution.

**Figure 5:**
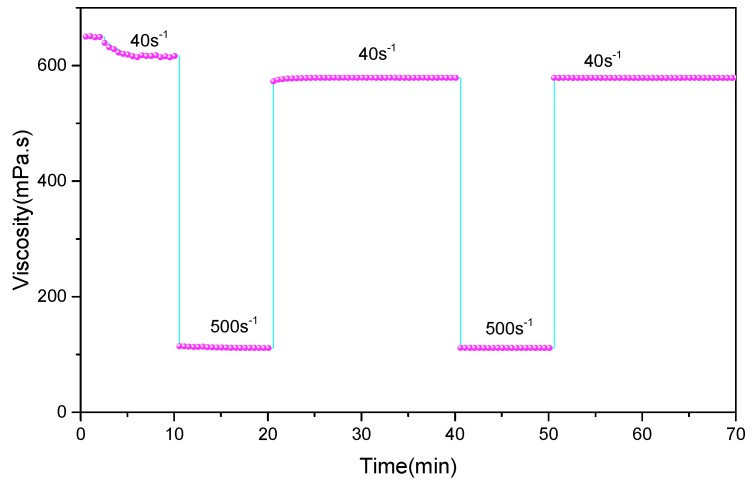
Shear recovery test of the 0.6 wt% PAANM solution.

The ESEM image (Figure 4) shows that the PAANM forms dense network structures in solution. Intermolecular chains and internal chains are connected by hydrophobic side chains, which make them have much higher viscosity and elasticity than ordinary linear polyacrylamides. Since the hydrophobic interaction is a kind of reversible physical interactions by self-aggregation, the spatial structure can quickly recover after being destroyed by shearing. As shown in Figure 5, the polymer solution exhibits good shear resistance due to the reversibility of the association. After shearing at the high rate of 500 s^−1^ for 10 min, the viscosity of the polymer solution rapidly restored to near the level at 40 s^−1^ before shearing and then remained constant. 

### 3.2. Effect of Incorporation of N-Vinyl-2-pyrrolidone (NVP) in PAANM on Its Temperature Resistance

It has been widely reported in the literature that NVP can be used to synthesize high temperature resistant water-soluble polymer [41,42,43,44] as the multi-membered ring structure of the NVP can enhance the rigidity of the main chains and temperature resistance [42,43]. Guo et al. (2011) modified hydroxypropyl guar gum by grafting rigid pyrrolidone groups and ester groups into the main chain and demonstrated improved high temperature resistance by rheological analyses and successful stimulation of a reservoir at 184.6 °C [45].

**Figure 6:**
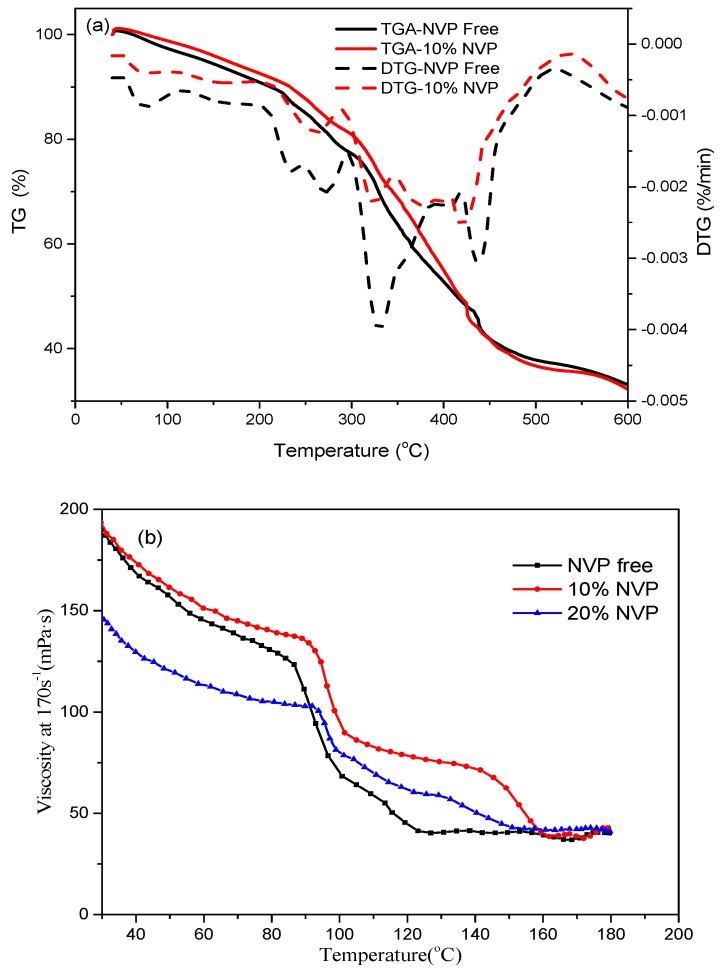
Effect of NVP on (**a**) thermal stability of polymer powders measured by TGA and DTG, and (**b**) viscosity of the polymer solutions (temperature ramp 3 °C/min).

TGA and derivative thermogravimetry (DTG) curves in Figure 6a showed that the NVP-containing and NVP-free HAWSP have similar temperature resistance. First, the temperatures at which both polymers start to degrade are around 210 °C. The high content of amide groups in both polymers makes them absorb moisture easily and the weight loss before 210 °C can be attributed to primarily water evaporation. Second, their semi-degradation temperatures are similar (around 420 °C). Third, although there are slight differences in local values, for example, the DTG in 300–400 °C, TGA and DTG have the same trend of change in the whole temperature range (30–600 °C). As fracturing fluids thickener, the working temperature of HAWSPs is usually below 200 °C. At this temperature, the polymer chain is relatively stable, and there is no obvious thermal degradation. Even if the temperature reaches 300 °C, the polymer containing NVP has no obvious advantage. Therefore, the incorporation of NVP does not improve the anti-thermal degradation ability of the polymer itself at its working temperature. 

As shown in Figure 6b, when 10% NVP was added, the initial viscosity of the polymer solution is similar to that of NVP free counterpart. However, the polymer solution’s temperature resistance has been significantly improved, especially in the temperature range of 90–160 °C. TGA analysis showed that NVP does not affect thermal stability. However, after adding NVP, the viscosity of solution at the high temperature is kept better. Therefore, we believe that NVP makes the polymer chain more rigid, the molecular thermal motion ability is weakened, and the thermal thinning characteristics are suppressed, so that the hydrophobic association structure is relatively better at high temperatures in solution [46]. When the temperature exceeds 160 °C, the molecular thermal motion is further increased, weakening the effect of NVP. So that the viscosity of the two polymers is almost the same.

When the NVP content increased to 20%, the initial viscosity decreased due to the lower degree of the polymerization caused by higher chain transfer constant of NVP. Even so, its viscosity is obviously higher than that of polymers without NVP between 90–150 °C. In addition, the polymer with the highest NVP content has the smallest total viscosity drop.

Therefore, by adding a certain amount of NVP, and at a certain temperature (90–160 °C), the viscosity of the polymer solution can be increased.

### 3.3. Effect of Deoxidizer on Temperature Resistance of PAANM (10% NVP) Solution

Dissolved oxygen in solution can break down polyacrylamide carbon-carbon bonds (or cause major chain scissions of polyacrylamide) at high temperature, resulting in reduced molecular weight of the polymer and degraded solution properties. Temperature stabilizers or deoxidizers are commonly used to solve this problem. For instance, by adding a certain amount of sodium thiosulfate into the traditional guar gum fracturing fluids, the viscosity of the fracturing fluids can be improved by 2 to 10 times at high temperature compared with that without using sodium thiosulfate or the fracturing fluids can resist temperatures that are 10–20 °C higher [47].

**Figure 7:**
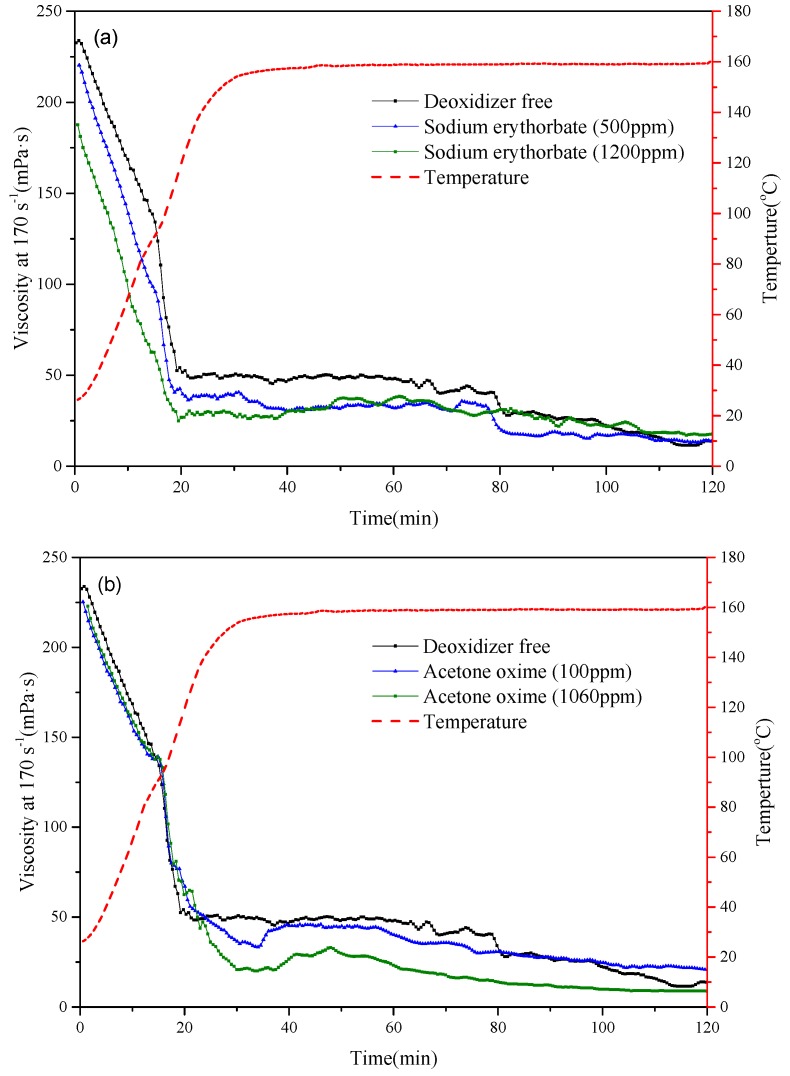

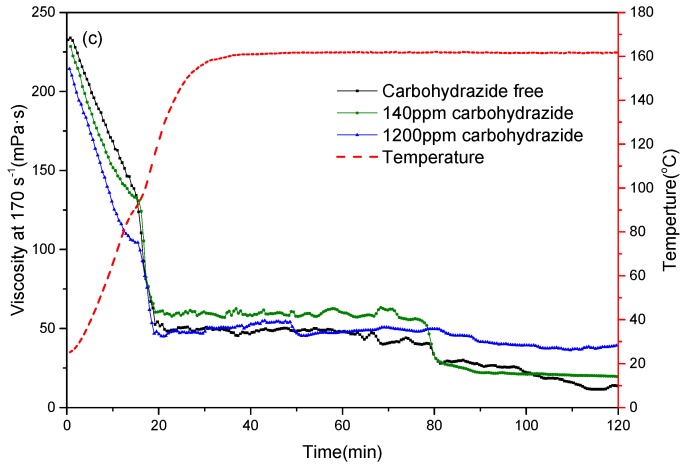
Effect of environmentally-friendly deoxidizer (**a**) acetone oxime; (**b**) sodium erythorbate; and (**c**) carbohydrazide on solution thermal stability of PAANM (10% NVP) solution.

For acrylamide-based polymers, compatible temperature stabilizers are important because improper temperature stabilizers may react with polymers and lead to faster degradation [12]. Therefore, deoxidizers were first screened, including acetone oxime, sodium erythorbate, and carbohydrazide (Figure 7a–c). Comparing with the deoxidizer-free solutions, acetone oxime and sodium erythorbate resulted in lower viscosity while carbohydrazide performed the best in maintaining the solution viscosity. It can be seen that with a concentration of 1200 ppm carbohydrazide, the solution viscosity leveled out during the whole testing period (Figure 7c).

### 3.4. Effect of Polyethyleneimine (PEI) on Temperature Resistance of PAANM (10% NVP) in the Presence of the Deoxidizer

Carbohydrazide could improve the temperature resistance of PAANM, making it possible to meet the industry standard. If it is used as fracturing fluids, it can already meet applications to formation temperature of 160 °C (viscosity is above 50 mPa·s at the shear rate of 170 s^−1^ and 160 °C in Chinese petroleum industry standard [48]). However, as temperature continues to increase, the viscosity could decrease below the industry standard. Therefore, to overcome the applicable temperature limit, PEI was added to form a new network structure when the association network was severely damaged. The crosslinking reaction between PEI and amide groups is shown in Figure 2 [27,49].

**Figure 8:**
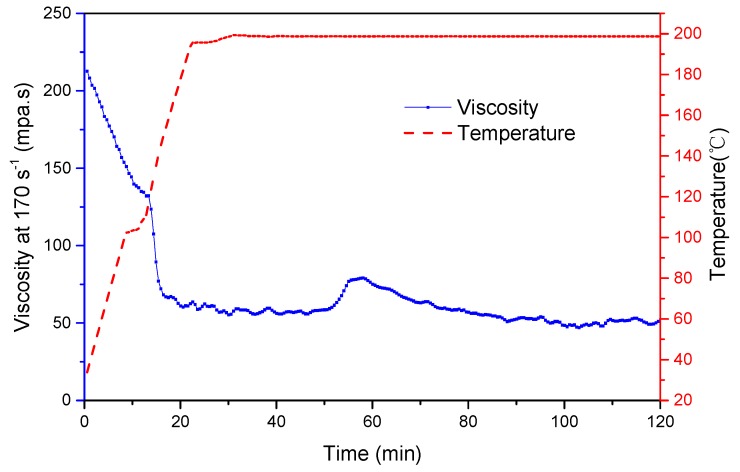
Viscosity vs. time of the 0.6 wt% PAANM (10% NVP) solution with 150 ppm of PEI and 1000 ppm of carbohydrazide.

As shown in Figure 8, after being heated for 50 min, the solution viscosity started to increase, indicating that the new networks began to form. When the PEI concentration reached 150 ppm, the fluids viscosity was maintained above 50 mPa·s at 200 °C and the constant shear rate of 170 s^−1^. Since PAANM is a kind of water-soluble and hydrophobically associative polymer, it is capable of maintaining a high viscosity itself (i.e., without being reacted with PEI). Therefore, the solution viscosity can stay above the industry standard during the entire testing process. The observation that the PAANM can maintain its viscosity higher than 50 mPa·s across the whole temperature range at 170 s^−1^ underlines the success of the triple-protection strategy. 

### 3.5. Performance Testing of the HAWSP as Fracturing Fluids

Hydraulic fracturing serves to form fractures in the formation through hydraulic transmission of high pump pressure, thus improving the flow capacity of oil and gas. In order to prevent the crack from closing after the pressure is released, proppant (quartz sand, ceramsite, etc.) must be filled in the crack. So high proppant carrying capacity is important for fracturing fluids, which can effectively transport the sand through long distance and eventually generate high permeability fracture networks. Otherwise, it may affect the hydraulic fracturing effect and even lead to operation failure.

**Figure 9:**
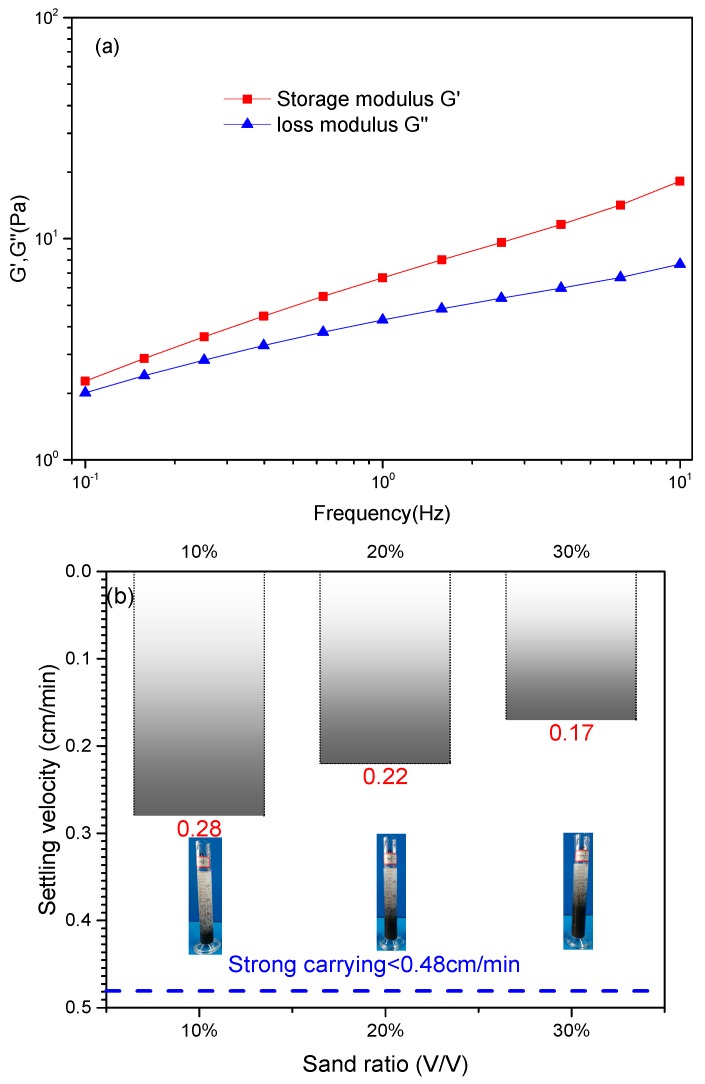
Performance as fracturing fluids at room temperature: (**a**) Storage modulus G’ and loss modulus G’’ of, and (**b**) settling velocity of the ceramsites in the 0.6 wt% PAANM solution with 150 ppm of PEI and 1000 ppm of carbohydrazide.

The proppant carrying capacity of viscous fluids mainly depends on their viscosity, for example, the guar gum fracturing fluids. But for viscoelastic fluids, such as viscoelastic surfactant solution, a lower viscosity could still meet the requirement of suspending proppant [6], due to their stronger structural elasticity. Thus, for viscoelastic fluids, the storage modulus G’ and the ratio of storage modulus to loss modulus (G’ / G’’) are both important parameters. Figure 9a shows that the storage modulus of 0.6 wt% PAANM solutions with 150 ppm of PEI and 1000 ppm of carbohydrazide is always higher than the loss modulus, indicating that the “triple-protection” PAANM solution is a kind of viscoelastic fluids with strong sand carrying capacity.

Measuring the settling velocity of sand in fracturing fluid is a simple method which can be used to evaluate the carrying capacity of a fracturing fluid. It is accepted that a settling velocity that is lower than 0.48 cm/min signifies strong sand carrying capacity of fracturing fluids [50]. The results in Figure 9b show that the settling velocities of ceramsite in the PAMNM fracturing fluids are below 0.48 cm/min regardless of the proppant concentration.

**Figure 10:**
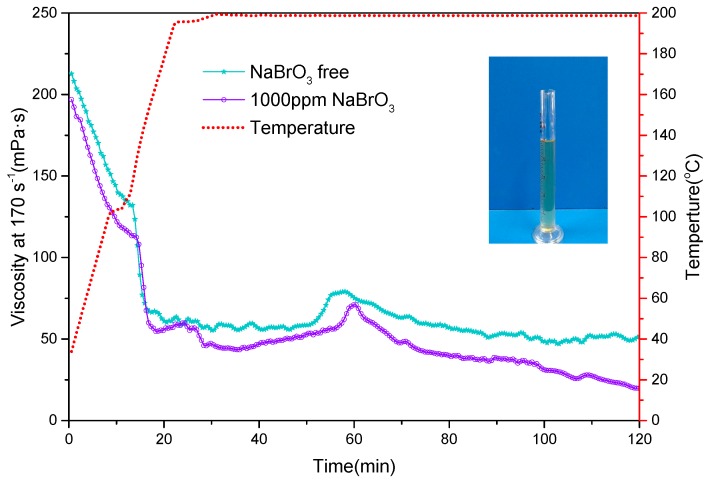
Viscosity profile of 0.6 wt% PAANM fracturing fluid with sodium bromate at 200 °C.

Polymers have to be degraded to allow them to flow back after fracturing, otherwise, they might block the formation and reduce the oil and gas productivity. Usually, polyacrylamide gels can be broken by oxidizers. Gupta and Carman (2011) reported the usage of sodium bromate as a breaker [25], which was tested with the PAANM fracturing fluids in this study. Figure 10 compares the viscosity profiles of the “triple-protection” PAANM solutions with and without sodium bromate at 200 °C. All broken fluids are clear and transparent without any gel residual and as an example, Figure 10 shows the picture of a broken fluid after being oxidized at 200 °C. This indicated that the “triple-protection” PAANM gel solution can be completely degraded and potential reservoir may be very low.

## 4. Conclusions

The viscosity of HAWSP solutions may decrease rapidly at high temperatures because of oxidative degradation and thermal movement. A comprehensive strategy was implemented to prepare high temperature resistant HAWSP fluids which can be used as fracturing fluids for high temperature applications, i.e., up to 200 °C. Based on a series of characterization experiments, the following conclusions can be drawn:

(1) The introduction of the rigid groups of NVP did not improve the resistance of HAWSP to thermal degradation, but increased its viscosity retention at elevated temperatures due to the increase rigidity of the polymer chain.

(2) The viscosity of HAWSP solution became more stable by adding a certain amount of carbohydrazide as the deoxidizer. 

(3) PEI is a high temperature response crosslinking agent that can remain inactive at low temperature but quickly crosslink HAWSP at high temperature to form networks. It can effectively compensate for the viscosity loss caused by thermal degradation. 

(4) HAWSP with good solubility and high temperature resistance was prepared.
